# *Bacillus firmus* Strain I-1582, a Nematode Antagonist by Itself and Through the Plant

**DOI:** 10.3389/fpls.2020.00796

**Published:** 2020-07-10

**Authors:** Zahra Ghahremani, Nuria Escudero, Daniel Beltrán-Anadón, Ester Saus, Marina Cunquero, Jordi Andilla, Pablo Loza-Alvarez, Toni Gabaldón, F. Javier Sorribas

**Affiliations:** ^1^Departament d’Enginyeria Agroalimentària i Biotecnologia, Universitat Politècnica de Catalunya, Barcelona, Spain; ^2^Bioinformatics and Genomics Program, Centre for Genomic Regulation (CRG), Barcelona Institute of Science and Technology, Barcelona, Spain; ^3^Department of Experimental and Health Sciences, Universitat Pompeu Fabra, Barcelona, Spain; ^4^Institut de Ciències Fotòniques (ICFO), The Barcelona Institute of Science and Technology, Barcelona, Spain; ^5^Institució Catalana de Recerca i Estudis Avançats, Barcelona, Spain

**Keywords:** *Cucumis sativus*, induced resistance, nematode antagonist, *Meloidogyne incognita*, root-knot nematodes, *Solanum lycopersicum*

## Abstract

*Bacillus firmus* I-1582 is approved in Europe for the management of *Meloidogyne* on vegetable crops. However, little information about its modes of action and temperature requirements is available, despite the effect of these parameters in its efficacy. The cardinal temperatures for bacterial growth and biofilm formation were determined. The bacteria was transformed with GFP to study its effect on nematode eggs and root colonization of tomato (*Solanum lycopersicum*) and cucumber (*Cucumis sativus*) by laser-scanning confocal microscopy. Induction of plant resistance was determined in split-root experiments and the dynamic regulation of genes related to jasmonic acid (JA) and salicylic acid (SA) by RT-qPCR at three different times after nematode inoculation. The bacteria was able to grow and form biofilms between 15 and 45°C; it degraded egg-shells and colonized eggs; it colonized tomato roots more extensively than cucumber roots; it induced systemic resistance in tomato, but not in cucumber; SA and JA related genes were primed at different times after nematode inoculation in tomato, but only the SA-related gene was up-regulated at 7 days after nematode inoculation in cucumber. In conclusion, *B. firmus* I-1582 is active at a wide range of temperatures; its optimal growth temperature is 35°C; it is able to degrade *Meloidogyne* eggs, and to colonize plant roots, inducing systemic resistance in a plant dependent species manner.

## Introduction

Plant parasitic nematode (PPN) management is a challenge for reducing crop yield losses all across the world. These parasites are responsible for annual yield losses reaching ca. 10% of life-sustaining crops and 14% of economically important crops (Agrios, 2005), varying greatly between areas due to specific nematode-plant-environment interactions. Root-knot nematodes (RKN) belonging to the genus *Meloidogyne* are the most damaging PPN worldwide due to their wide range of plant hosts, worldwide distribution and high reproductive capacity (Jones et al., 2013). The potential damage of some *Meloidogyne*-crop combinations has been summarized by Greco and Di Vito (2009). Currently, RKN management strategies tend to reduce dependence from chemical nematicides by encouraging alternative control methods that promotes both the incompatible plant-RKN relationship by the use of plants bearing resistance genes (*R*-genes) and/or by microbe-inducing plant resistance, and the antagonistic potential of soils against the nematode.

During the course of a compatible infection, second-stage juveniles (J2) of *Meloidogyne* enter the root near the elongation zone and migrate intercellularly to circumvent the endodermal barriers and establish a permanent feeding site into the vascular cylinder, which induces the formation of giant cells and root galls. The J2 secrete effectors that affect cell wall architecture, plant development, plant defenses and metabolism to complete their life cycle successfully (Shukla et al., 2018). Subsequently, J2 become sedentary and molt three times before achieve the mature adult female stage. The most frequently encountered and damaging tropical species, *M. arenaria, M. incognita*, and *M. javanica*, reproduce parthenogenetically. The female lays a large number of eggs in a gelatinous matrix, known as the egg mass, located on the surface or inside galled roots. In an incompatible plant-RKN relationship due to plant *R*-genes, expression of plant genes related to phytohormone-mediated resistance responses, signal transduction pathways and secondary metabolite-mediated resistance responses are activated leading to suppression of nematode development and reproduction (Shukla et al., 2018). Plant hormones play a regulatory role in the induction of plant resistance by microorganisms (Pieterse et al., 2014). Primed plants can improve resistance to stressing agents during their life cycle and the effects can be maintained over generations (Mauch-Mani et al., 2017).

The antagonistic potential of a soil is the capacity to reduce the spread of deleterious agents to plants through biotic factors (Sikora, 1992). Several nematode antagonists can occur naturally in agricultural soils providing some degree of suppressiveness (Giné et al., 2016; Elhady et al., 2017). High levels of soil suppressiveness can be achieved under favorable interactions between plant-RKN-antagonists, cultural practices, and abiotic factors (Giné et al., 2016). In soils with no or low levels of soil suppressiveness, nematode antagonists can be introduced by the application of biological-based nematicides. Currently, three biological-based nematicides are approved in Europe: *Bacillus firmus* I-1582 (Bf I-1582), *Purpureocillium lilacinum* (= *Paecilomyces lilacinus*) strain 251 and *Pasteuria nishizawae* Pn1. Among them, only Bf I-1582 and *P. lilacinum* strain 251 are approved for use against RKN in vegetable crops (European Commission, 2019).

Vegetables are important components of the human diet and are cultivated worldwide in both open field and under protected structures. Rotation sequences including fruiting solanaceous and cucurbit crops are very common because they represent the main source of income for many growers. Unfortunately, all of them are affected by RKN (Hallman and Meressa, 2018). Some attempts to manage RKN using *P. lilacinum* strain 251 have shown a high percentage of fungal egg parasitism in both *in vitro* and pot experiments (Khan et al., 2004, 2006; Kiewnick and Sikora, 2006a, b; Kiewnick et al., 2011). However, limited efficacy have been reported in field experiments under Mediterranean conditions (Anastasiadis et al., 2008; Kas̨kavalci et al., 2009; Giné and Sorribas, 2017) probably due to sustained sub-optimal soil temperatures experienced during the cropping seasons and/or the inhibition of fungal enzymes by components of the formulation (Giné and Sorribas, 2017). In the case of Bf I-1582, some reports have shown its effectivity against several PPN including RKN under different experimental conditions, from *in vitro* to field experiments on different crops (Giannakou et al., 2004, 2007; Mendoza et al., 2008; Terefe et al., 2009; Castillo et al., 2013). This bacterial strain reduced egg hatching and the viability of nematodes in *in vitro* experiments, and suppressed nematode reproduction and disease severity in pot and field experiments. Moreover, Bf I-1582 triggered systemic resistance in soybean (*Glycine max*) infected with *Heterodera glycines*, and in cotton (*Gossypium hirsutum*) parasitized by RKN in split-root experiments, but not in corn (*Zea mays*) infected with *M. incognita* (Schrimsher, 2013; Gattoni et al., 2018). The interactions of *Bacillus* species and plants with special reference to induced systemic resistance (ISR) have been reviewed (Choudhary and Johri, 2009). It seems that specific transduction pathways promoted during ISR by *Bacillus* spp. vary depending on the bacterial strain, the host plant and the plant pathogen (Kloepper et al., 2004). Extensive work has been done with several *Bacillus* spp., but information about *B. firmus* in particular remained limited until 2013 (Wilson and Jackson, 2013). Since then, novel information concerning nematicidal virulence factors (Geng et al., 2016; Zheng et al., 2016; Marin-Bruzos and Grayston, 2019) and induction of plant resistance in cotton (Gattoni et al., 2018, 2019) has been reported.

Optimal use of microbial antagonists against RKN on vegetable crops cultivated under irrigation has to consider the cardinal temperatures of microbial growth, the effect on the most abundant and vulnerable nematode development stages, and the plant-microbe interaction, especially the putative induction of plant resistance against nematodes. In this manuscript, all these aspects are presented: (i) cardinal temperatures for Bf I-1582 growth and biofilm formation; (ii) effect of Bf I-1582 transformed with green fluorescent protein gene (Bf I-1582 -GFP) on RKN eggs; (iii) colonization of tomato and cucumber roots by Bf I-1582 -GFP; and (iv) putative induction of systemic resistance and the dynamic regulation of genes related to the jasmonic acid (JA) and salicylic acid (SA) pathways in tomato (*Solanum lycopersicum*) and cucumber (*Cucumis sativus*).

## Materials and Methods

### Sources of Planting Material and Nematode and Bacteria Inoculum

Tomato cv. Durinta and cucumber cv. Dasher II, both susceptible to RKN (Giné et al., 2016) were used in this study. For all the experiments seeds were surface-sterilized in a 50% bleach solution (40 g l^–1^ NaOCl) for 2 min, washed three times in sterilized distilled water for 10 s each. The seeds were then sown in a tray containing sterile vermiculite and maintained in a growth chamber at 25°C ± 2°C with a 16 h:8 h (light:dark) photoperiod.

The *Meloidogyne incognita* isolate Agropolis used in this study came from a nematode population obtained from tomato cv. Durinta roots cultivated in a plastic-greenhouse in 2010 in which tomato and cucumber were previously cultivated. Twenty tomato plants cv. Durinta grown in 200 cm^3^ pots filled with sterile sand were inoculated with a single egg mass each and maintained in a growth chamber at 25 ± 2°C and 16 h:8 h photoperiod for 45 days. The adult females developing from a single egg mass were identified as *M. incognita* by the morphology of perineal pattern and by SCAR markers (Zijlstra et al., 2000). The offspring were maintained on tomato cv. Durinta for experimental purposes. J2 were used as inoculum. Eggs were extracted from tomato roots by blender maceration in a 5% commercial bleach (40 g l^–1^ NaOCl) solution for 10 min (Hussey and Barker, 1973). The egg suspension was filtered through a 74 μm aperture sieve to remove root debris, and eggs were collected on a 25 μm sieve and placed on Baermann trays (Whitehead and Hemming, 1965) at 25°C ± 2°C. J2 were collected daily using a 25 μm sieve for 7 days and stored at 9°C until use.

The Bf I-1582 strain isolated from the VOTiVO^®^ formulation (Bayer CropScience) was used in the cardinal temperatures and biofilm formation experiments, as well as for the bacterial transformation with the green fluorescent protein (GFP) gene to study plant root and nematode egg interactions. The commercial formulate VOTiVO^®^ was used in the induction of plant resistance experiments and gene expression analysis.

### Bf I-1582 GFP Transformation

Bf I-1582 was transformed with the GFP gene with a pAD43-25 plasmid (Dunn and Handelsman, 1999) kindly provided by the *Bacillus* Genetic Stock Center. Bacterial protoplasts were prepared according to Aono et al. (1992) with slight modifications. Three ml of Bf I-1582 cells grown overnight in Penassay medium (1.5 g l^–1^ beef extract, 1.5 g l^–1^ yeast extract, 5 g l^–1^ peptone, 1 g l^–1^ glucose, 3.5 g l^–1^ NaCl, 3.6 g l^–1^ dipotassium phosphate and 1.32 g l^–1^ monopotassium phosphate) were harvested by centrifugation at 3000 g for 10 min at 4°C. The pellet was suspended in 0.1 ml of SMMP medium consisting of SMM (0.5M sucrose, 0.02 M maleic acid and 0.02 M MgCl_2_) in double-strength Penassay media pH 6.4, supplemented with 40 mg of lysozyme, and incubated at 37°C with gentle shaking for 75 min. The *B. firmus* protoplasts were recovered by centrifugation at 1000 g for 30 min at 10°C, washed twice with SMMP medium and finally suspended in 0.1 ml of SMMP media.

Transformation of Bf I-1582 was performed according to Chang and Cohen (1979). Briefly, 150 ng of purified plasmid pAD43-25 were mixed with 5 μl of 2X strength SMM buffer and 50 μl of *B. firmus* protoplasts obtained as explained above. Then, 150 μl of 40% polyethylene glycol 8000 (Scharlau) were added, and 0.5 ml of SMMP media were added after 2 min of incubation. Protoplasts were recovered by centrifugation at 2500 rpm for 10 min. Protoplasts were suspended in 0.1 ml of SMMP media and kept at 30°C with gentle shaking for 4 h. Finally, cells were plated in solid Luria-Bertani (LB) supplemented with 20 μg ml^–1^ chloramphenicol and incubated at 37°C. After 2 days, GFP expression of pAD43-25 of the transformed Bf I-1582 colonies was assessed in a Hertel and Reuss CN-hF microscope with appropriate filters.

To corroborate that the species transformed with the pAD43-25 plasmid was *B. firmus*, one stable colony transformant was selected. This colony was inoculated in LB and growth O/N before performing a genomic DNA extraction and a PCR with BLS342F (5′ CAGCAGTAGGGAATCTTC 3′) and 1392R (5′ ACGGGCGGTGTGTACA 3′) primers following the conditions described in Blackwood et al. (2005). The 1050 bp PCR product obtained was sequenced and aligned against nr using nBLAST with default settings.

### Live-Cell Imaging of Tomato and Cucumber Roots and *M. incognita* Eggs Colonized by Bf I-1582-GFP

Tomato and cucumber seeds were surface-sterilized, as described previously, and were placed on a moist chamber at 25°C until the main root was ca. 2 cm long (5–7 days). Seedlings were transferred to an Erlenmeyer flask filled with 80 ml of 0.5X Murashige and Skoog (MS) semisolid agar medium (0.07% agar) and inoculated with 20 ml of the Bf I-1582-GFP overnight culture grown at 35°C in liquid LB supplemented with 20 μl ml^–1^ chloramphenicol (Sigma). Seedlings were incubated at 25°C for 5 and 10 days, respectively (Hao and Chen, 2017). Afterward, roots were washed with distilled water to eliminate non-adhered bacteria and 10 fragments of the root system (ca. 1 cm long) were examined with laser-scanning confocal microscopy. To determine the spatial pattern of root colonization by Bf I-1582-GFP, roots were imaged using a Leica TCS 5 STED CW microscope (Leica Microsystem) equipped with hybrid detectors and with a 40x 1.25NA HCX Pl Apo CS Leica objective. A 488 nm laser was used for fluorescence excitation. GFP fluorescence was detected at 505–530 nm and autofluorescence of root cell walls at 580–620 nm, as described in Macià-Vicente et al. (2009). Stacks of 8–13 μm, step size of 0.2–0.3 μm, were acquired. Z projection-images and Z stack movies are shown at Figure 2 and Supplementary Video S1, respectively. All image analysis was performed using Fiji (Schindelin et al., 2012).

Nematode eggs were surface-sterilized as in McClure et al. (1973). A 200 μl suspension containing ca. 100 eggs were dispensed in a 1.5 ml microcentrifuge tube containing 50 μl of bacterial culture grown on LB supplemented with 20 μg ml^–1^ chloramphenicol (Sigma) at 35°C for 2 days. After 3, 5 and 10 days of incubation at 35°C ± 2°C eggs were examined with laser-scanning confocal microscopy using a Leica TCS 5 STED CW microscope (Leica Microsystem) equipped with hybrid detectors and with a 63x 1.4NA HCX Pl Apo CS Leica objective. A 488 nm laser was used for fluorescence excitation and transmission-light detection. GFP fluorescence was detected at 505–530 nm and egg autofluorescence was detected at 580–620 nm (Escudero and Lopez-Llorca, 2012). Stacks of 8–13 μm, step size of 0.2–0.3 μm, were acquired. Z projection-images are shown in Figure 3. A three-dimensional (3D) reconstructed Z-stack (Supplementary Video S2) of an *M. incognita* egg after 10 days from bacterial inoculation was created using Huygens software (Huygens SVI, Netherlands).

### Cardinal Temperatures of Bf I-1582 and Biofilm Formation

Cardinal temperatures of Bf I-1582 were determined. Sets of three Petri dishes containing nutrient agar (3 g l^–1^ beef extract, 5 g l^–1^ peptone, 5 g l^–1^ NaCl, 15 g l^–1^ agar, pH 7) were inoculated with ca. 200 bacteria colony-forming units (CFU) and incubated in the dark at 4, 9, 20, 25, 30, 35, 40, 45, and 50°C for 96 h before counting.

Growth kinetics in liquid media were determined inoculating 10^6^ CFU in Erlenmeyer flasks containing 200 ml of LB. Sets of three Erlenmeyer flasks were incubated at 10, 15, 20, 25, 30, 35, 40, 45, and 50°C. Cultures were maintained with shaking and one aliquot of 3 ml was taken at 0, 2, 4, 6, 8, 10, 12, 24, 30, 36, and 48 h to measure the optical density at 590 nm (OD_590 nm_) in an UV-Vis Evolution 300 spectrophotometer (Thermo Fisher Scientific, United States).

Sets of three Petri dishes (40 mm diameter) with 10 ml of Schaeffer’s sporulation medium plus glucose and glycerol (SGG) (5 g l^–1^ beef extract, 10 g l^–1^ peptone, 2 g l^–1^ KCl, 0.5 g l^–1^ MgSO_4_ 7H_2_O, 1 mM Ca(NO_3_)_2_, 0.1 mM MnCl_2_⋅4 H_2_O, 1 μM FeSO_4_, 1 g l^–1^ glucose, and 1% glycerol) were inoculated with 10^6^ CFU and incubated at 10, 15, 20, 25, 30, 35, 40, 45, and 50°C in the dark for 2 days to determine the effect of the temperature on the Bf I-1582 biofilm formation (Hsueh et al., 2015).

### Induction of Plant Resistance by Bf I-1582 Against *M. incognita*

Tomato and cucumber were grown in a split-root system as described in previous studies (Ghahremani et al., 2019). In this system, the main root is divided into two halves transplanted in two adjacent pots: the inducer, inoculated with Bf I-1582 and the responder inoculated with 200 J2 of *M. incognita*. Briefly, the main root of 5-day-old seedlings was excised and plantlets were individually transplanted into seedling trays containing sterile vermiculite and maintained in a growth chamber at 25 ± 2°C and 16 h:8 h photoperiod for 3 weeks for tomato and 2 weeks for cucumber. Afterward, plantlets were transferred to the split-root system by splitting roots into two halves planted into two adjacent 200 cm^3^ pots filled with sterilized sand. The experiment consisted of three treatments: Bf I-1582/*M. incognita* (Bf-Mi) and *M. incognita* (None-Mi) to assess the capability of Bf I-1582 to induce plant resistance against *M. incognita*, and a control non-inoculated with neither bacteria or nematode to assess the effect of the split-root system on each root half development. Each treatment consisted of ten individual plants. Two experiments were conducted with tomato, and one with cucumber.

The inducer pot was inoculated with a liquid suspension of 10^9^ CFU of BF I-1582 one week after transplantation. One week after bacterial inoculation, the responder pot was inoculated with a liquid suspension to achieve a soil density of 1 J2 of *M. incognita* per cm^3^. The treatment None received the same volume of water. The plants were maintained in a growth chamber under the same conditions described previously in a completely randomized design for 43 days. Plants were irrigated as needed and fertilized with Hoagland solution twice per week. Soil temperatures were recorded daily at 30 min intervals with a PT100 probe (Campbell Scientific Ltd.) placed in the pots at a depth of 4 cm. At the end of the experiment, plants were uprooted and fresh root weight of both inducer and responder halves from the non-inoculated control treatment were measured. Roots from responder pots inoculated with *M. incognita* were immersed in a 0.01% erioglaucine solution for 45 min to stain the egg masses (Omwega et al., 1988) before counting them. Afterward, the nematode eggs were extracted from the roots according to the Hussey and Barker (1973) procedure in a 10% commercial bleach solution (40 g l^–1^ NaOCl) for 10 min and counted.

Endophytic root colonization by Bf I-1585 was estimated at the end of the first and second experiment with cucumber and tomato, respectively. Bacterial DNA was quantified by qPCR from three individual biological samples from the Bf-Mi treatment. Each composite biological sample consisted of the inducer half of the roots from three plants. For the composite inducer sample, half of the roots (of three plants) were surface sterilized using 50% commercial bleach solution (40 g l^–1^ NaOCl) for 2 min and washed three times with sterile distilled water for 10 s each, and then blotted onto sterile paper. The DNA was extracted from each biological replicate following the López-Llorca et al. (2010) procedure. The qPCR reactions were performed using the Brilliant Multiplex QPCR Master Mix (Agilent Technologies) in a final volume of 25 μl containing 50 ng of total DNA and 0.5 μM of each primer (5′–3′ direction): Votivo-2F (forward) CTCCAATTCCTAATATCCTGCAAAG, Votivo-2R (reverse) GGAAAGTCACGGGACAGTTAT (Mendis et al., 2018). Negative controls containing sterile water instead of DNA were included. Reactions were performed in duplicate in a Stratagene Mx3005P thermocycler (Agilent Technologies) using the following thermal cycling conditions: initial denaturation step at 95°C for 15 min, then 39 cycles at 95°C for 30 s, and 58°C for 1 min. DNA of Bf I-1582 was used to define a calibration curve ranging from 5 pg to 50 ng. PCR specificity was verified by means of melting curve analysis and agarose gel electrophoresis. The Bf DNA referred to the total DNA biomass (50 ng) is expressed as a proportion.

### Dynamic Expression of JA and SA Related Genes by Bf I-1582 and *M. incognita* in Tomato and Cucumber

Two-week-old cucumber seedlings and 3-week-old tomato seedlings were transferred to 200 cm^3^ pots filled with sterilized sand, and maintained in a growth chamber as previously described. The assessed treatments were: non-inoculated plants (Control), plants inoculated with Bf I-1582 (Bf), plants inoculated with *M. incognita* (Mi) and plants co-inoculated with both organisms (Mi+Bf). Bf I-1582 treatments were inoculated with 10^9^ CFU 1 week after transplantation, and those *M. incognita* treatments were inoculated with 200 J2 2 weeks after transplanting. The expression of the genes related to JA and SA pathways was evaluated at 0 days after nematode inoculation (DANI), at 7 DANI, when the nematode infected the roots, and at 40 DANI. Root infection was determined by staining the nematode into the root with acid fuchsin (Byrd et al., 1983). At 40 DANI, the nematode eggs were extracted by the Hussey and Barker (1973) from roots of three individual tomato or cucumber plants from all inoculated treatments. Total tomato and cucumber root colonization by Bf I-1585 was estimated from three different plants of each Bf treatment by qPCR at 0 and 40 DANI. Roots were washed three times in sterilized distilled water for 10 s each and then blotted onto sterile paper. The DNA extraction and qPCR were conducted as described under section “Induction of Plant Resistance by Bf I-1582 Against *M. incognita*.”

For the expression study, three biological replicates were assessed at each sampling time. Each biological replicate consisted of a composite sample of roots from three plants that were pooled. Roots excised from the aboveground plant parts were washed three times with sterile distilled water, blotted on sterile filter paper, and immediately frozen with liquid nitrogen. Samples were stored at −80°C until used. RNA isolation and retrotranscription were conducted according to Ghahremani et al. (2019). Dynamic regulation in the JA pathway was determined by the expression of the lipoxygenase D (*LOX-D*) gene for tomato (Fujimoto et al., 2011) and lipoxygenase 1 (*LOX1*) gene for cucumber (Shoresh et al., 2004). In the SA pathway we evaluated the expression of the pathogenesis-related 1 (*PR1*) gene for tomato (Gayoso et al., 2007) and the phenylalanine ammonia-lyase (*PAL*) gene for cucumber (Shoresh et al., 2004). The ubiquitin (*UBI*) gene was used as a reference gene for both plant species (Yang et al., 2012; Song et al., 2015). Relative gene expression was estimated with the 2(-Delta Delta C(T)) method (Livak and Schmittgen, 2001). The sequences of the primers used in the RT-qPCR are shown in Supplementary Table S1. The qPCR reactions were performed in a final volume of 20 μl with 1 μl of cDNA, 0.3 mM primers and 1X Fast SYBR Green Master Mix (Applied Biosystems) in a 7900HT Fast Real Time PCR System thermocycler (Applied Biosystems). Reactions were performed with two technical replicates per each biological replicate using the following conditions: 20 s at 95°C followed by 40 cycles of 30 s at 95°C and 1 min at 60°C for tomato (Gayoso et al., 2007) and 20 s at 95°C followed by 40 cycles of 15 s at 95°C and 1 min at 60°C for cucumber (Shoresh et al., 2004). PCR specificity was verified by means of melting curve analysis and agarose gel electrophoresis.

### Statistical Analysis

Statistical analyses were performed using the JMP software v8 (SAS institute Inc., Cary, NC, United States). Both data normality and homogeneity of variances were assessed. When confirmed, a paired comparison using the Student’s *t*-test was undertaken. Otherwise, paired comparisons were conducted using the non-parametric Wilcoxon test (*P* < 0.05).

## Results

### Cardinal Temperatures of Bf I-1582 and Biofilm Formation

**FIGURE 1:**
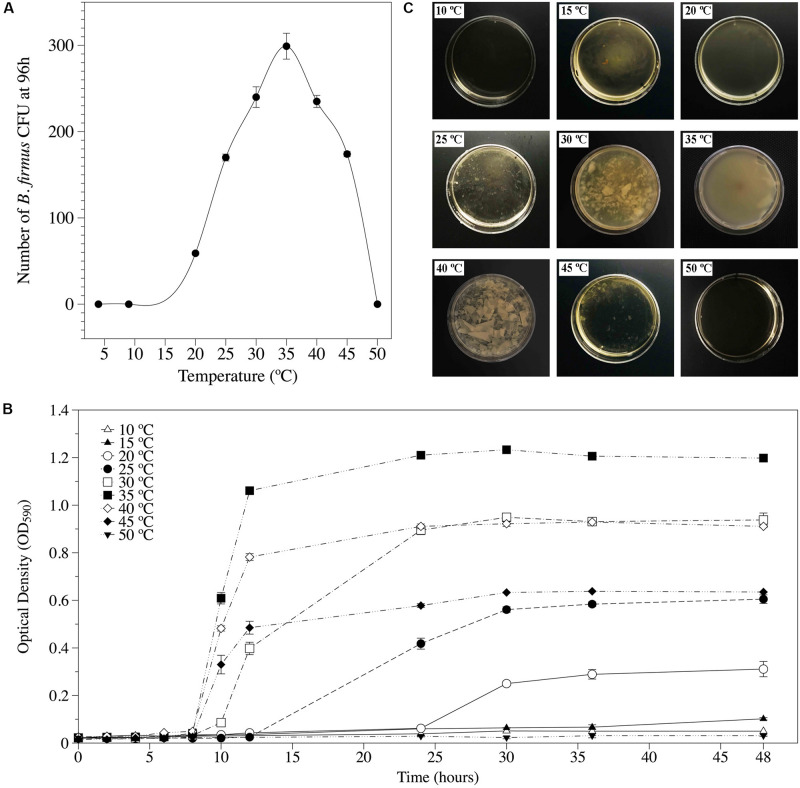
Cardinal temperatures for *Bacillus firmus* I-1582 growth and biofilm formation. **(A)** Number of bacterial CFU after 96 h of incubation in solid nutrient agar at different temperatures. Each value is mean ± standard error of three replicates. **(B)** Bacterial growth kinetics incubated for 0, 2, 4, 6, 8, 10, 12, 24, 30, 36, and 48 h in Luria-Bertani (LB) liquid media at different temperatures. Each value is mean ± standard error of three replicates. **(C)** Biofilm formation in Schaeffer’s sporulation medium plus glucose and glycerol (SGG) media 48 h after being inoculated with 10^6^ bacterial CFU and incubated at different temperatures.

Bf I-1582 grew in temperatures ranging from 15 to 45°C, with 35°C being the optimal growth temperature in both solid (Figure 1A) and liquid media (Figure 1B). Similarly, biofilm formation was observed between 15 and 45°C, being thicker and uniform at 35°C (Figure 1C).

**FIGURE 2:**
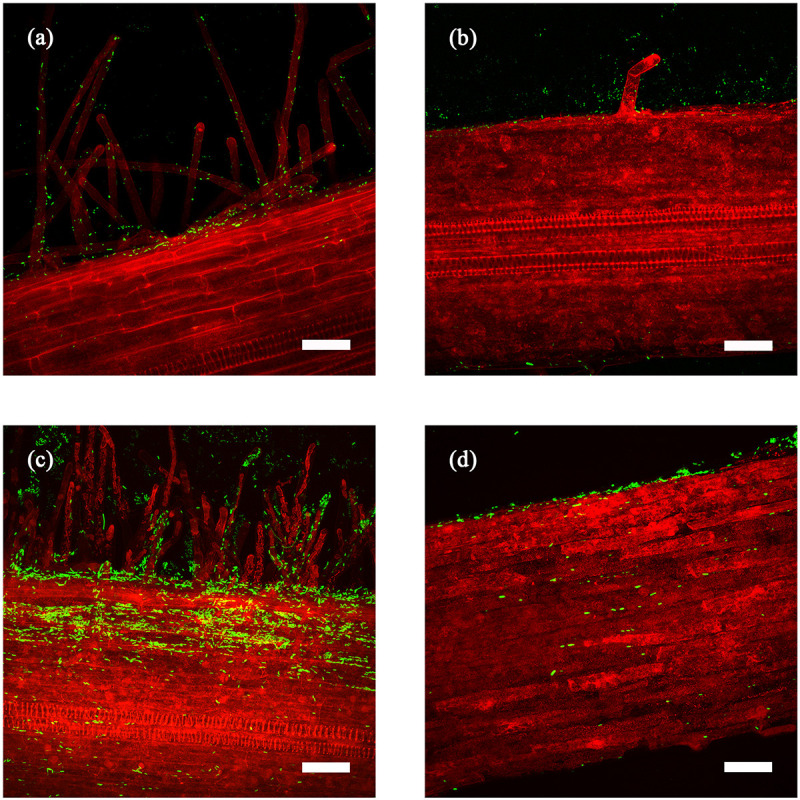
Z projection of laser-scanning confocal microscopy images of *Bacillus firmus* I-1582 transformed with the green fluorescent protein gene (Bf I-1582GFP) colonizing tomato **(a,c)** and cucumber **(b,d)** roots after 5 **(a,b),** and 10 **(c,d)** days after bacterial inoculation and incubation at 25°C. Scale bar: 50 μm. Autofluorescence of roots is shown in red.

**FIGURE 3:**
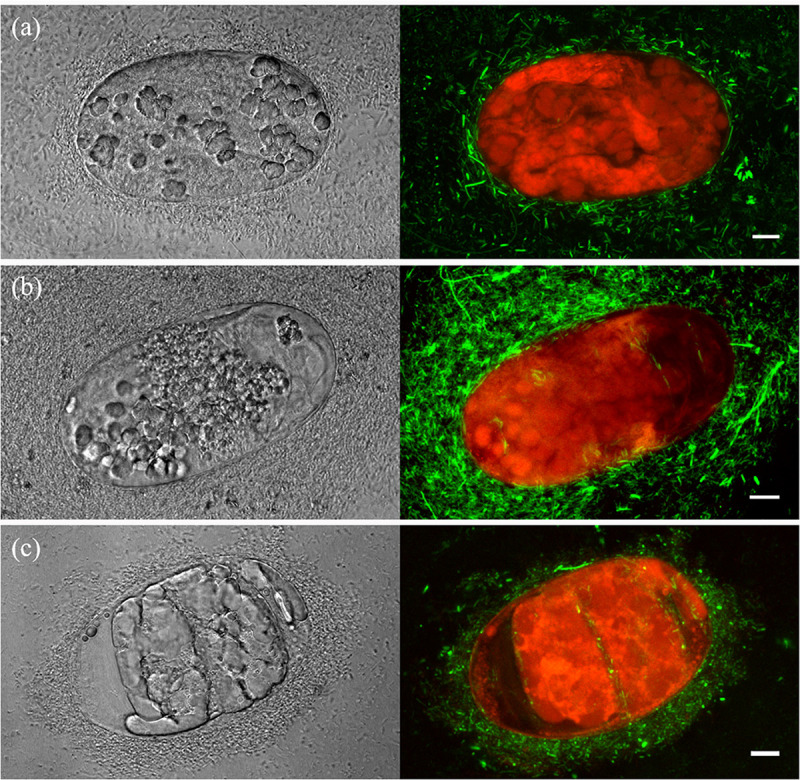
Laser-scanning confocal microscopy images of *Bacillus firmus* I-1582 transformed with the green fluorescent protein gene (Bf I-1582GFP) damaging *Meloidogyne incognita* eggs after 3 **(a)**, 5 **(b),** and 10 **(c)** days of incubation at 35°C. Z projection images of transmitted (left) and fluorescence (right) channels. Scale bar: 10 μm. Autofluorescence of nematode eggs is shown in red.

### Tomato and Cucumber Roots Colonization and *M. incognita* Eggs Degradation by Bf I-1582-GFP

Both tomato and cucumber roots were colonized by *B. firmus* (Figure 2). In tomato, Bf I-1582GFP was observed on root hairs and epidermal cells at 5 days after inoculation (DAI) (Figure 2a). At 10 DAI bacterial colonies were observed in root hairs and some lone bacteria were found inside the root (Figure 2c). In cucumber, few bacteria were observed on epidermal cells at 5 DAI (Figure 2b). There were no bacteria found inside the root at 10 DAI (Figure 2d) with the few cells observed being attached to the surface of the root section, as shown in Supplementary Video S1.

*M. incognita* egg-shell degradation and egg colonization by Bf I-1582-GFP were studied with confocal-scanning laser microscopy (Figure 3). At 3 DAI, bacteria were surrounding and degrading the nematode egg and embryo (Figure 3a); at 5 DAI, bacterial colonies were adhered to the egg-shell and some bacteria were found inside the egg (Figure 3b); at 10 DAI bacterial biofilms adhered to the egg-shell and bacteria inside the egg were observed (Figure 3c). Egg-shell erosion and egg colonization by Bf I-1582-GFP can be observed in the Supplementary Video S2.

### Bf I-1582 Induces Plant Resistance to *M. incognita* in Tomato but Not in Cucumber

Fresh root weight of the two halves of the non-inoculated split-root system did not differ significantly (*P* < 0.05; data not shown) in either tomato or cucumber, proving that the treatment did not hamper root development.

**FIGURE 4:**
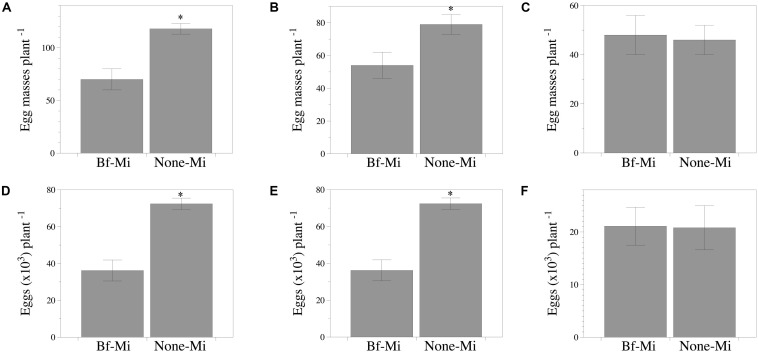
Capability of *Bacillus firmus* I-1582 to induce systemic resistance in tomato cv. Durinta and cucumber cv. Dasher II against *Meloidogyne incognita* in two split root experiments. The inducer part of the root was inoculated with 10^9^ CFU of *B. firmus* I-1582 and the responder part of the root was inoculated with 200 J2 of *M. incognita*. In the responder part of the root the number of egg masses per plant (infectivity) and the number of eggs per plant (reproduction) were assessed 43 days after nematode inoculation. Number of egg masses per plant **(A–C)** and total nematode eggs per plant **(D–F)**. Tomato experiment 1 **(A,D)**; tomato experiment 2 **(B,E)**; cucumber experiment **(C,F)**. Data are means ± standard error of 10 replicates. The asterisk indicates within each graph and for each experiment, that treatments are significantly different (*P* < 0.05) according to the non-parametric Wilcoxon test.

Lower number (*P* < 0.05) of egg masses and eggs per plant were recorded in the responder part of the tomato root of the Bf-Mi treatment compared to the None-Mi, irrespective of the experiment (Figures 4A,B,D,E). However, no effect was detected in cucumber (Figures 4C,F).

**FIGURE 5:**
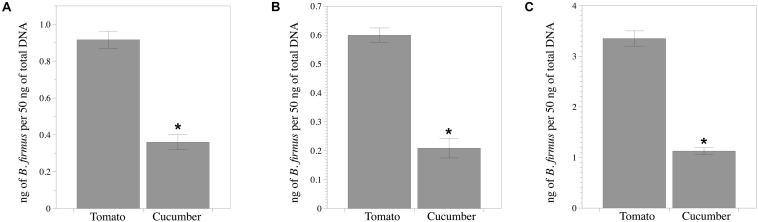
*Bacillus firmus* I-1582 colonizes tomato and cucumber roots. **(A)** Endophytic bacterial DNA biomass in relation to the total DNA biomass of the sample from the inducer half of the root 50 days after being inoculated with 10^9^ CFU and 43 days after nematode inoculation with 200 *Meloidogyne incognita* juveniles in the responder half of the root of the split-root experiment. The inducer part of the root was surface sterilized with 50% commercial sodium hypochlorite for 2 min and washed three times with sterile distilled water for 10 s each prior to the DNA extraction. Each value is mean ± standard error of three biological replicates composed of three plant roots each. **(B)** Bacterial DNA biomass in relation to the total DNA biomass of the sample 7 days after bacterial inoculation at a rate of 10^9^ CFU per plant and just after nematode inoculation with 200 J2 (0 DANI) and **(C)** at 40 DANI. Each value is mean ± standard error of three biological replicates composed of one plant root each. Asterisk indicates significant difference between plant species according to the non-parametric Wilcoxon test (*P* < 0.05).

The standard curve for qPCR used for estimating the bacterial DNA density was: Ct = −3.1413 ^∗^ log10 DNA concentration + 24.522 (*R*^2^ = 0.9591). *B. firmus* colonized roots of both plant species endophytically, but ca. 61% higher (*P* < 0.05) density of bacterial DNA was recorded in tomato than in cucumber roots (Figure 5).

### Dynamic Regulation of JA and SA Genes by Bf I-1582 Is Plant-Dependent

**FIGURE 6:**
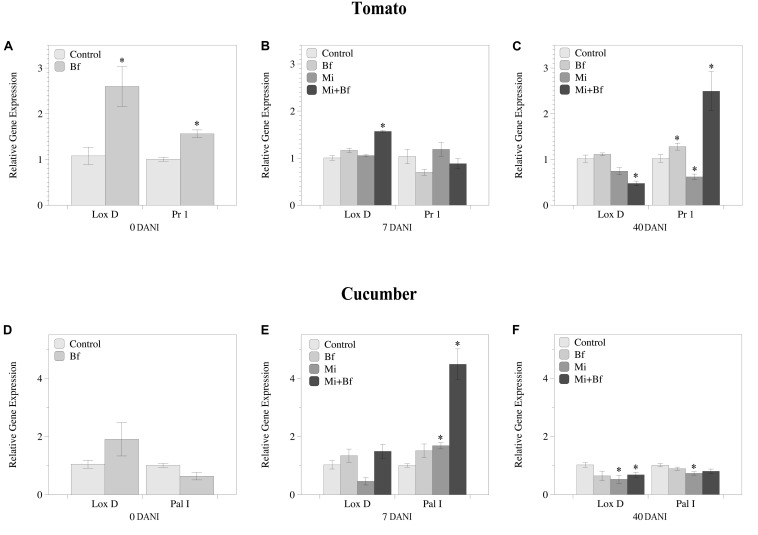
Dynamics of the relative gene expression of genes related to the jasmonic acid (*Lox D*) and the salicylic acid (*PR1/Pal I*) pathways in tomato **(A–C)** and cucumber **(D–F)** plants that were non-inoculated (Control), inoculated with *Bacillus firmus* I-1582 (Bf), inoculated with *Meloidogyne incognita* (Mi) and co-inoculated with both nematode and bacteria (Mi+Bf). Bf I-1582 treatments were inoculated with 10^9^ CFU 1 week after transplanting, while *M. incognita* treatments were inoculated with 200 juveniles 2 weeks after transplanting. The relative expression of the genes was evaluated at 0 days after nematode inoculation (DANI), at 7 and at 40 DANI. The ubiquitin (*UBI*) gene was used as a reference gene for both plant species. Each value is mean ± standard error of three biological samples with two technical replicates each. Each biological replicate consisted of the roots from three plants pooled together. Asterisk indicates significant differences with respect to the Control using the non-parametric Wilcoxon test (*P*< 0.05).

The regulation of genes related to JA and SA pathways varied between tomato and cucumber (Figure 6). In tomato, at 0 DANI, which corresponds to 7 days after Bf I-1582 inoculation and just after nematode inoculation, both JA (*LoxD*) and SA (*Pr1*) pathways were up-regulated in plants inoculated with the bacteria in comparison to non-inoculated plants (Figure 6A). At 7 DANI, when nematode infection was established, only the plants co-inoculated with nematode and bacteria showed an up-regulation of the JA related gene (*Lox D*) (Figure 6B). At 40 DANI, when J2 hatching began and new root infections occurred, tomato plants co-inoculated with the nematode and Bf I-1582 had repressed the JA related gene (*LoxD*). Meanwhile the gene related to the SA pathway (*Pr1*) was up-regulated in plants co-inoculated and also inoculated with Bf I-1582 alone, but was suppressed in plants inoculated only with the nematode (Figure 6C).

In cucumber plants, no differences between treatments were found at 0 DANI (Figure 6D). At 7 DANI *Pal I* was up-regulated both in the *M. incognita* inoculated plants and those co-inoculated with the bacteria and the nematode (Figure 6E). At 40 DANI, both JA and SA pathways were suppressed in plants inoculated with *M. incognita* but only JA in the co-inoculated plants (Figure 6F).

**FIGURE 7:**
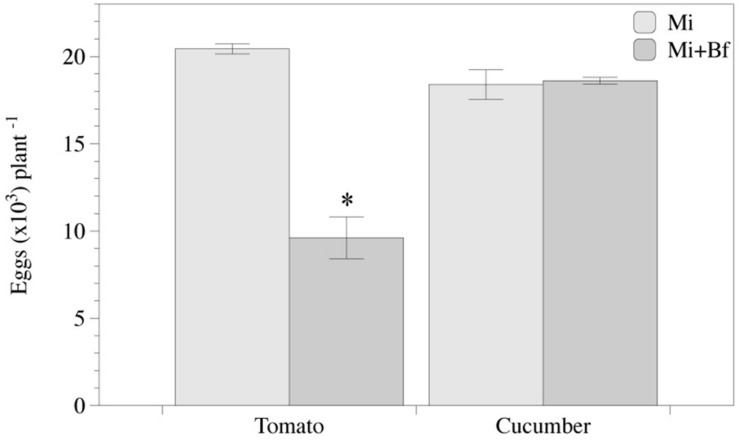
*Meloidogyne incognita* reproduction is affected in *Bacillus firmus* I-1582 primed tomato plants but not in cucumber. Number of eggs in tomato and cucumber plants 40 days after inoculation with *M. incognita* (Mi) or co-inoculated with 10^9^ CFU of Bf I-1582 one week after transplanting and with 200 juveniles of *M. incognita* 2 weeks after transplanting (Mi+Bf). Each value is mean ± standard error of three replicates. Asterisk indicates significant differences between treatments per each plant species according to the non-parametric Wilcoxon test in tomato or Student’s *t*-test in cucumber (*P*< 0.05).

Nematode reproduction in tomato co-inoculated with bacteria was reduced (*P* < 0.05) in a 53%, but did not differ between treatments in cucumber. Bacterial colonization of tomato roots was ca. 65% higher in tomato than in cucumber at 0 and 40 DANI (Figure 7).

## Discussion

The results of this study contribute to better understanding of *B. firmus-*tomato or cucumber interactions, two economically important fruiting vegetables cultivated worldwide; and *B. firmus-Meloidogyne* eggs interaction, the most abundant nematode developmental stage in soil and in plant roots. In addition, determining the cardinal temperatures of bacterial growth and biofilm formation provide valuable information to optimize the use of *B. firmus*–based formulations to maximize nematode control efficacy.

*B. firmus* was able to colonize both tomato and cucumber roots, but it grew more efficiently on tomato. The GFP-transformed bacteria colonized the rhizoplane of both plant species, and the observation of attenuated fluorescence indicates that some bacteria could colonize tomato roots endophytically. This finding was corroborated by qPCR, showing that the proportion of bacteria inside or on the surface of tomato roots was ca. 61–65% higher than that found inside or on cucumber roots. It is known that *B. firmus* is able to colonize roots of other economically important crops such as corn, soybean and cotton (Mendis et al., 2018; Gattoni et al., 2019). Root colonization by bacteria could prevent nematode infection and production of viable inoculum by affecting the nematode cuticle and the egg-shell. Some reports pointed out the capability of *B. firmus* to inhibit J2 hatching, motility and viability (Mendoza et al., 2008; Terefe et al., 2009; Xiong et al., 2015; Gattoni et al., 2018). In the current study, the degradation of nematode eggs by the GFP-transformed bacteria was observed by laser-scanning confocal microscopy. Serine proteases have been reported in several fungal and bacterial nematode antagonists as a key factor affecting directly the plant-parasitic nematode physical barriers (Bonants et al., 1995; Segers et al., 1996; Lian et al., 2007; Iqbal et al., 2018). Recently, Geng et al. (2016) identified the serine protease Sep 1 from *B. firmus* DS-1, which is capable of degrading multiple cuticle and intestinal-associated proteins. Thus, bacterial colonies growing on or inside the roots could affect the nearest infective or sedentary nematodes and eggs by some of the nematicidal virulence factors exhibited by *B. firmus* (Geng et al., 2016; Zheng et al., 2016; Marin-Bruzos and Grayston, 2019). Further investigation of this type of interaction is required.

In the present study, Bf I-1582 induced systemic resistance against *M. incognita* in tomato but not in cucumber in split-root experiments. Previous experiments showed that this bacterial isolate was able to induce systemic resistance in cotton cv. Phytogen 333 WRF (Gattoni et al., 2018) but not in an unspecified corn cultivar (Schrimsher, 2013). Thus, these results support the hypothesis that this phenomenon is plant species dependent at least, because all these studies were conducted using only one cultivar of each plant species. Further studies should be conducted to determine the ability of Bf I-1582 to induce resistance in a range of cultivars from economically important crops to determine its putative use to manage RKN and other PPN through the plant. In our study, the relative expression of genes related to SA and JA pathways was assessed in tomato and cucumber at 7 days after bacterial inoculation and just after nematode inoculation (0 DANI), after root infection by the nematode (7 DANI), and when the offspring reinfected plant roots (40 DANI). We observed some differences between plant species. Tomato plants were primed by SA and JA at 0 DANI, and the nematode infection was reduced, measured as the number of egg masses recorded at the end of the split-root experiment. However, no differences in the expression of SA and JA related genes was observed in cucumber plants inoculated with bacteria as well as in the number of egg masses produced in cucumber roots. Martinez-Medina et al. (2017) and Ghahremani et al. (2019) considered that SA primed plants affect nematode infection, corroborating the results of the tomato experiments conducted in this study. At 7 DANI, *Lox D*, gene related with JA biosynthesis was up-regulated in tomato and could affect nematode development and reproduction, as proposed by Martinez-Medina et al. (2017) and Ghahremani et al. (2019). Nonetheless, *PAL*, a key regulatory enzyme in the synthesis of SA that can be activated by the JA/ethylene pathway (Martinez et al., 2001; Shoresh et al., 2004) was up-regulated in cucumber in treatments inoculated with *M. incognita*. Our results are in concordance with those reported by Shukla et al. (2018) who observed *PAL* up-regulation during disease development in tomato plants inoculated with *M. incognita*. In cucumber, no effect on nematode infection and/or nematode reproduction was observed at the end of the split-root or co-inoculation experiments. Time elapsed between bacterial and nematode inoculations could explain this delay in plant response, since bacterial colonization was less efficient in cucumber than in tomato roots, as assessed by laser-scanning confocal images and qPCR measuring of bacterial DNA. At 40 DANI, the SA related genes were down-regulated in both tomato and cucumber plants inoculated only with the nematode agreeing with previous results reported by Shukla et al. (2018). However, it was up-regulated in tomato plants co-inoculated with the bacteria and the nematode, according to the incompatible nematode-plant interaction (Shukla et al., 2018) while no effect was observed in cucumber. Regarding the JA related gene, it was down-regulated in both plant species co-inoculated with the bacterium and the nematode. The dynamic regulation of genes related to SA and JA induced by *B. firmus* I-1582 and *B. amyloliquefaciens* QST713 in cotton against *M. incognita* has been studied recently (Gattoni et al., 2019). At 1 week after inoculation, the gene related to SA was up-regulated by both bacterial strains, and the authors proposed that a long-term SA-dependent systemic response was responsible for nematode suppression. According to our results, *B. firmus* I-1582 induces a response in plants but its effects on nematodes vary depending on the host plant species. In plant species for which the nematode is suppressed, a shift from SA to JA regulation genes affects nematode infection and reproduction, as it has been reported for *Trichoderma harzianum*-tomato (Martinez-Medina et al., 2017) and *Pochonia chlamydosporia*-tomato interactions (Ghahremani et al., 2019). Conversely, regulation of plant defense genes can also occur but at a time at which no effect on nematodes is observed, as we found for cucumber. The result of the *B. firmus*-cucumber interaction could be explained by the influence of root exudates, such as *p*-coumaric acid, on the growth of the bacteria in the rhizosphere, in turn affecting the concentration of bacterial compounds, such as surfactin, reported as plant resistance elicitors (Cawoy et al., 2014; Zhou et al., 2018).

The results of our study have shown that *B. firmus* I-1582 is a nematode antagonist that can act by itself and through the plant by induction of plant defenses, a mechanism that is plant species dependent. These findings are particularly interesting for the development of strategies aimed to maximize the efficacy of *B. firmus* I-1582-based formulations against RKN. Indeed, the application of formulates to the substrate of tomato plants seven days before transplantation will allow root colonization and early induction of resistance to nematode infection and reproduction. Moreover, the bacterium could also act by itself reducing nematode egg viability and consequently the potential of inoculum. In this regard, even if the bacterium would not have an effect on cucumber, it might have a direct effect when applied to the soil. In Greece, broad application of the *B. firmus* formulate significantly suppressed the number of females in roots and disease severity and/or J2 in soil after cropping cucumber in greenhouses from May to September at environmental temperatures ranging from 20 to 45°C (Giannakou et al., 2004). Giannakou et al. (2007) assessed the efficacy of the bacterial formulate alone and in combination with soil solarization against *M. incognita*. The number of nematodes in roots and the galling index of the following cucumber crop were reduced in *B. firmus* I-1582 treated plots compared to the control, but these parameters were higher than in plots treated with the chemical nematicide Basamid^®^ (active ingredient: dazomet). Nonetheless, no differences were found between plots treated with the chemical nematicide and the application of the bacterial strain just after soil solarization for 30 days with soil temperatures ranging from 32 to 40.5°C at 15 cm depth. In a pot experiment, the viability of J2 and eggs was suppressed after daily exposure at 35 and 40°C for 4 h followed by 20 h at 27–30°C for 2 and 4 weeks, respectively. In our study, the optimal temperature for bacterial growth and biofilm formation was recorded at 35°C. Thus, the application of the bacteria in summer when soil temperatures are around the optimal for bacterial growth and biofilm formation, will be the best time for nematode management in soil, reducing nematode densities and consequently the disease severity and crop yield losses.

## Conclusion

In conclusion, *B. firmus* I-1582 is a nematode antagonist able to degrade and colonize *Meloidogyne* eggs by itself, and also able to induce plant systemic resistance. However, this second effect varies depending on the host plant. This bacterial isolate is active across a wide range of temperatures, with an optimum of 35°C. This temperature is suboptimal for *Meloidogyne*, which could further enhance its antagonistic activity. Additional studies are required for maximizing its antagonistic potential and to design successful RKN management strategies, such as the bacterial capability to induce resistance in other important vegetable crops frequently included in rotation sequences; the putative effect of the induced resistance against virulent nematode populations to selected *R-*genes, as observed with the additive effects of resistance induced by *Trichoderma asperellum* isolate T34 in tomato with the Mi1.2 gene against a virulent *M. incognita* population (Pocurull et al., 2020) and the optimal timing for *B. firmus* I-1582-based formulations application under field conditions, among others.

## Data Availability Statement

All datasets generated for this study are included in the article/Supplementary Material.

## Author Contributions

FS and NE conceived, designed, supervised the experiments, the data collection, and analyses. ZG performed the experiments, analyzed the data, and wrote the draft of the manuscript. NE and DB-A transformed the bacterial strain. NE, MC, JA, and PL-A performed the nematode-bacteria and plant-bacteria laser-scanning confocal microscopy study. NE, ES, and ZG performed the molecular analysis. TG provided the reagents, materials, and advice. NE, ES, MC, PL-A, TG, and FS wrote the final version of the manuscript. All authors edited and approved the final manuscript.

## Conflict of Interest

The authors declare that the research was conducted in the absence of any commercial or financial relationships that could be construed as a potential conflict of interest.
