# Supplementary material for: Bacillus firmus Strain I-1582, a Nematode Antagonist by Itself and Through the Plant
Source: Front Plant Sci. 2020 Jul 10;11:796. doi: 10.3389/fpls.2020.00796 (PMC7381289; doi:10.3389/fpls.2020.00796)
Supplement: Supplementary file 1 [file Table_1.docx]

**Table S1** Primers used to quantify expression of genes related to jasmonic acid and salicylic acid pathways using RT-qPCR.

| Plant | Primers | Primer sequence (5’-3’) | PCR product size (pb) | Reference |
| --- | --- | --- | --- | --- |
| Tomato | LeUbi3-F LeUbi3-R | TCCATCTCGTGCTCCGTCT GAACCTTTCCAGTGTCATCAACC | 144 | Song *et al*. (2015) |
| Cucumber | UBQ-F  UBR-R | GGTGCCAAGAAGCGTAAGAA  CACCAGCTTTGTTGTAAACGT | 260 | Yang *et al*. (2012) |
| Tomato | LoxD-F  LoxD-R | GACTGGTCCAAGTTCACGATCC  ATGTGCTGCCAATATAAATGGTTCC | 178 | Fujimoto *et al.* (2011) |
| Cucumber | Lox1-F  Lox1-R | AAGGTTTGCCTGTCCCAAGA TGAGTACTGGATTAACTCCAGCCAA | 201 | Shoresh *et al*. (2004) |
| Tomato | LEPR1F  LEPR1R | GCAACACTCTGGTGGACCTT  ATGGACGTTGTCCTCTCCAG | 272 | Gayoso *et al*. (2007) |
| Cucumber | Pal1-R  Pal1-F | CCATGGCAATCTCAGCACCT  ATGGAGGCAACTTCCAA | 201 | Shoresh *et al*. (2004) |
|  |  |  |  |  |
